# Supplementary material for: Multi-Marker Immunofluorescent Staining and PD-L1 Detection on Circulating Tumour Cells from Ovarian Cancer Patients
Source: Cancers (Basel). 2021 Dec 10;13(24):6225. doi: 10.3390/cancers13246225 (PMC8699768; doi:10.3390/cancers13246225)
Supplement: Supplementary file 1 [file cancers-13-06225-s001.zip › cancers-1472442-supplementary.pdf]

Supplementary Materials

1. Methodology

1.1. MCF-7 as Negative Control Cell for PAX8

The breast cancer cell line MCF-7 was used as the negative control cell line for the PAX8 expression during the optimization process.

1.2. CD16 and CD45 Antibody Staining

Whole blood samples from healthy volunteers were enriched for WBCs using the Parsortix. The enriched cellular fractions were cytospun onto glass slides and stained. Briefly, enriched cells on glass slides were first permeabilised and blocked with 3% BSA, 0.2% Triton X-100, PBS, 1 M glycine, 10% NDS, FcR blocking reagent solution for 15 minutes at room temperature. Cells were then incubated for 60 minutes under room temperature using antibodies consisting Phycoerythrin (PE) labelled anti-CD45 (BD Biosciences) and Alexa Fluor 647 labelled anti-CD16 (Biolegend). Cells were washed with 1% BSA-PBS and then finally with PBS. The slides were then mounted using DAPI mountant, Fluoromount-G™, with DAPI (Thermo Fisher Scientific), overnight.

1.3. DAPI Mountants and Wet Mounting

Prolong Gold Anti-Fade reagent with DAPI (Thermo Fisher Scientific) in our laboratory, was initially used for the mounting. This was less water soluble, and thus did not aid in the efficient removal of the cover slip for the next round of fluorescence quenching. The DAPI mountant, Fluoromount-G™, with DAPI (Thermo Fisher Scientific) was also tested as used earlier for the quenching method (Adams et al., 2017; Adams, Alpaugh, Tsai, Tang, & Stefansson, 2016). Wet mounting using PBS was employed to improve efficiency of the method and to enable immediate or same day imaging of stained slides.

1.4. Fluorescent Quenching Effect on SKOV-3 Cell Lines

Quenching of fluorescently conjugated antibodies was optimised using the IFN-γ induced SKOV-3 cell lines. These cells express most of the biomarkers (CK/EpCAM, PD-L1, PAX8 and vimentin) used for detecting and characterizing the CTCs enriched in the ovarian cancer patients blood.

1.5. White Blood Cell Count after Parsortix Enrichment

White blood cell (WBC) count was done for each normal healthy control and patients' blood sample after Parsortix enrichment. These are all CD16/45 expressing cells after immunostaining.

1.6. Vascular Endothelia/CD31 Expressing Cells

Frequency of the CD31 positive cells, and the co-expression of the CTC markers were evaluated (Table 1) for the sixteen (16) CTC^{pos} patients and the 5 female healthy volunteer's enriched samples.

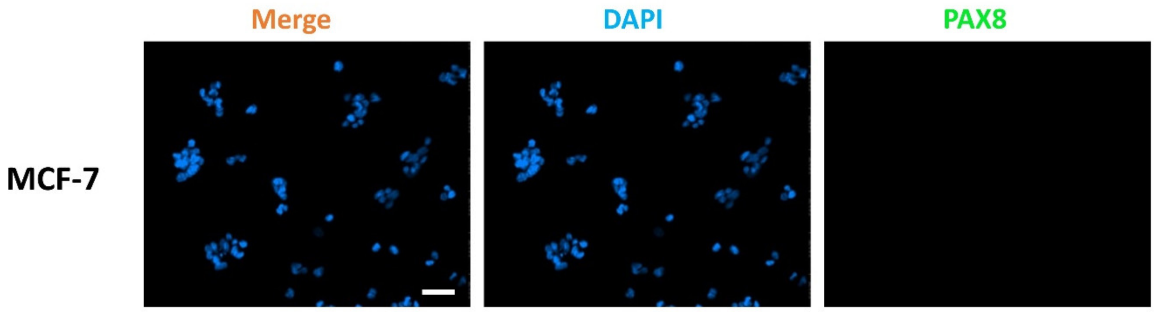

Figure S1. Micrograph showing MCF-7 showing no expression of the nuclei marker PAX8. PAX8 antibody conjugated with AF488. Scale bar = 50μm.

1.7. CD16 and CD45 Antibody Staining

From observed results from this study, the use of only CD45, provides staining of 60-80% of WBCs in Parsortix processed blood samples, as this process isolates larger WBCs such as granulocytes which express low levels of CD45. Thus, we decided to bolster WBC identification with the addition of CD16. Both CD16 and CD45 are WBC markers, however, they can be differentially expressed on sub-populations of these leucocytes. CD45 is not expressed on natural killer cells and is only weakly expressed on granulocytes, while expression of CD16 on both granulocytes and natural killer cells is higher (Cherian et al., 2010). The addition of CD16 to CD45 improved the WBC staining coverage than their individual use (Figure S2).

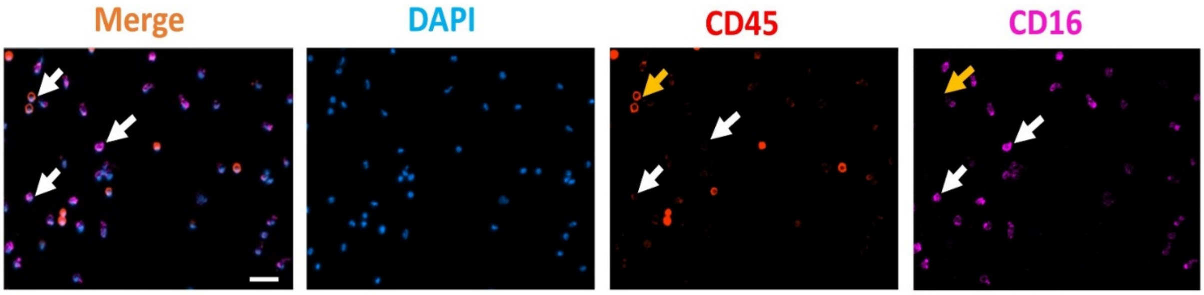

Figure S2. Photomicrograph depicting WBCs stained with CD45 conjugated to PE (red) and CD16 conjugated to Cy5 (purple). Both the CD16 and CD45 antibodies were diluted 1/50. Arrows identify cells with questionable/negative CD45 staining and strong CD16 staining. Scale bar = 50μm.

1.8. Comparing the use of DAPI mountants and wet mounting

Previously (before using PBS for the wet mounting), the Prolong Gold Anti-Fade reagent with DAPI (Thermo Fisher Scientific) in our laboratory, was initially used for the mounting. Unfortunately, this mountant could not be dissolved easily by PBS, and thus made the removal of cover slips from previously stained slides to be difficult. Furthermore, due to its poor solubility, it was not easily washed off from cells on slides when using PBS, and thus, could potentially prevent the effective quenching of the fluorophores and also could mask the epitopes of the cells preventing binding of antibodies during the second antibody staining (Figure S3). We then resorted to a more water soluble DAPI mountant, Fluoromount-G™, with DAPI (Thermo Fisher Scientific), which was used for the quenching method described earlier (Adams et al., 2016). It allowed for good staining of the cells on the quenched slides (Figure S4) and aided in the easy removal of the cover slips.

However, both mountants, whether highly water soluble or not, require the use of coverslips and their subsequent removal before quenching. The time taken to wash mountants off, together with the incubation period of mountants before microscopy, makes the entire process longer, as reported earlier (Adams et al., 2016). The above demerits of the DAPI mountants, paved the way for using the alternative wet mountant for the quenching method, as described in this current study.

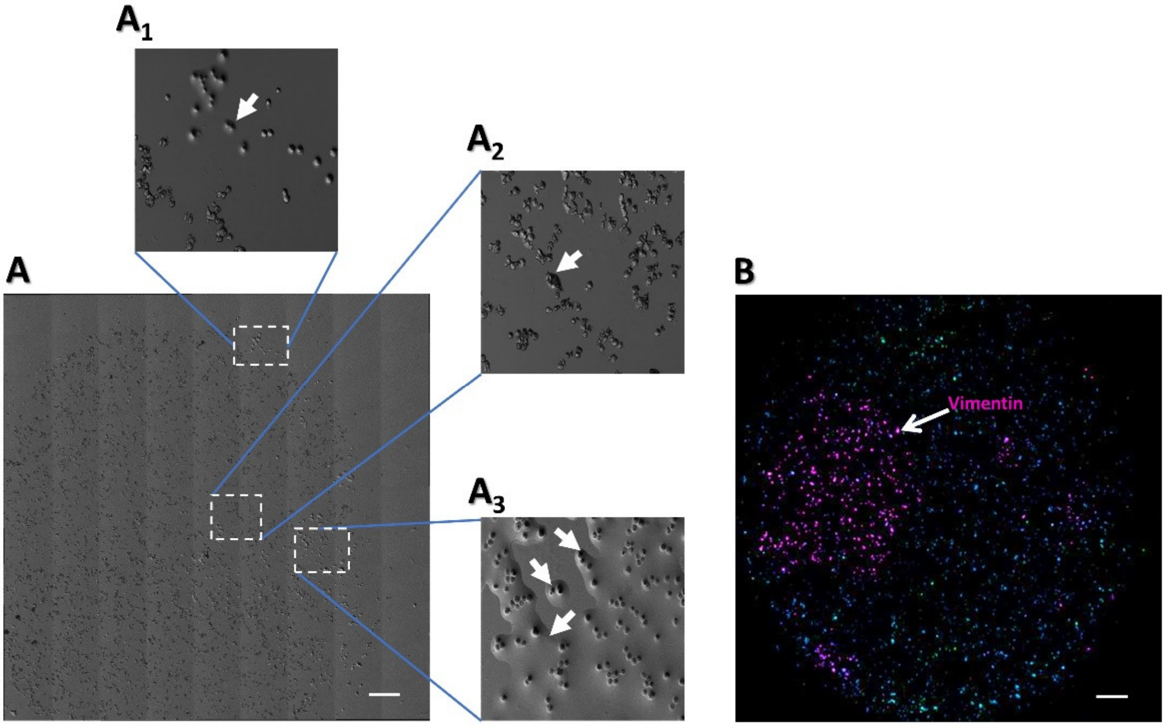

Figure S3. Photomicrograph of a slide with SKOV-3 cells after fluorescent quenching and re-staining with vimentin using the prolong Gold™ mountant. (**A**) Shows a bright-field micrograph, showing areas where mountants were not sufficiently or incompletely cleared, magnified in A_1, A_2, and A_3 (short white arrows). (**B**) This produced a patchy staining effect of the vimentin antibody staining (long white arrow) used in the second-round. Inefficient cytokeratin fluorescent (green colour) quenching can be seen on some surrounding cells. Scale Bar = 1 mm.

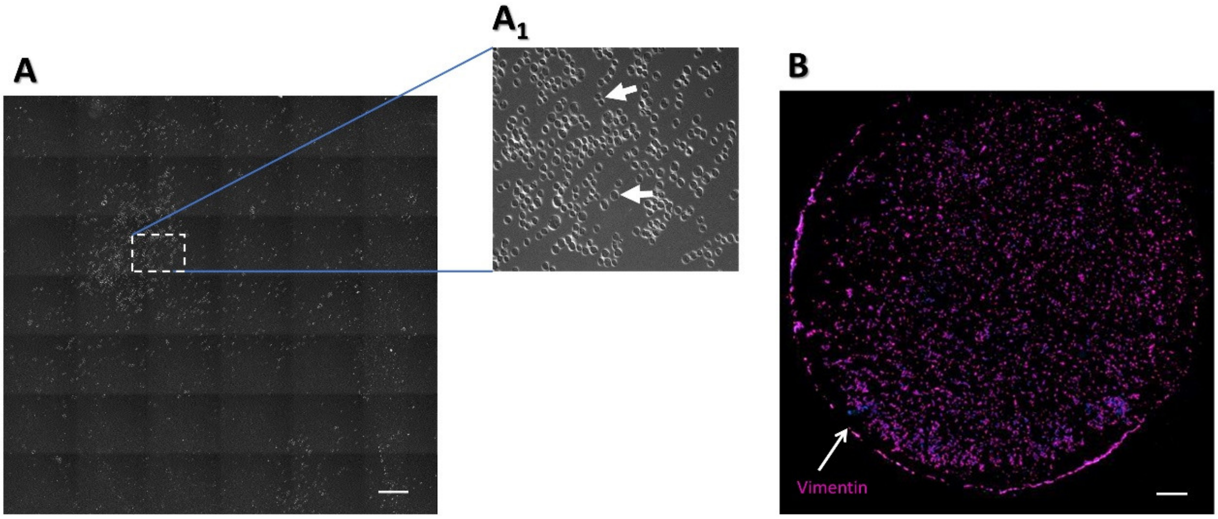

Figure S4. Photomicrograph of a slide with SKOV-3 cells after fluorescent quenching and re-staining with vimentin and subsequently mounting with Fluoromount Gold™ with DAPI. (**A**) Shows a bright-field micrograph, showing a completely cleared, montants in A_1 (short white arrows). This allowed for a uniform and even vimentin staining (long arrow) used in the second-round (**B**). Scale Bar = 1 mm.

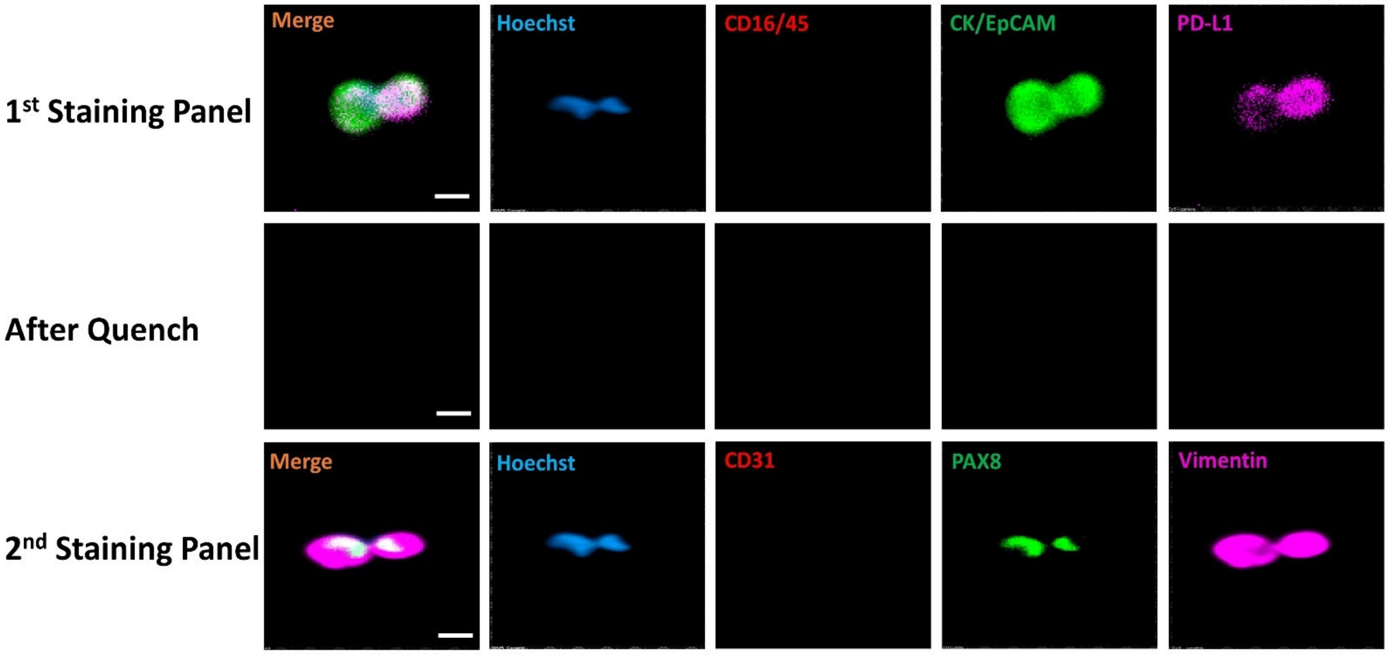

Figure S5. Photomicrograph showing evidence of quenching the 1st fluorescent markers (CK/EpCAM and PD-L1) and re-staining post-quenching with new markers (PAX8 and Vimentin). Scale Bar = 10 μm.

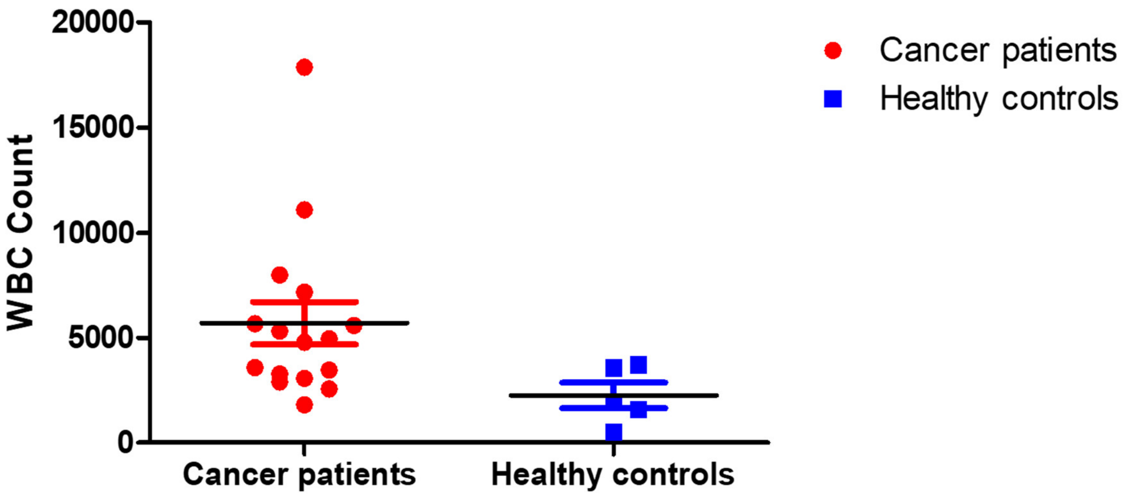

Figure S6. WBC count in cancer patients and healthy female blood donors. WBCs in the ovarian cancer patients (red) had a mean value of 5685 (range: 1813-17850 WBCs). The healthy controls on the other hand (blue), had mean WBC count of 2255 = (Range: 503-3698 WBCs), after enrichment with the Parsortix system.

Table S1. Frequency of detected CD31^{pos} cells and co-expression of the CTC markers in the OC patients and female healthy volunteers.

Number	ID	CD31	CK/EpCAM	Vimentin	PAX8	PD-L1
1.	OC 714	0	0	0	0	0
2.	OC 1248	0	0	0	0	0
3.	OC 1251	1	1	1	0	1
4.	OC 1313	0	0	0	0	0
5.	OC 1350	0	0	0	0	0
6.	OC 1354	0	0	0	0	0
7.	OC 1362	0	0	0	0	0
8.	OC 1409	3	3	1	0	3
9.	OC 1418	0	0	0	0	0
10.	OC 1423	7	0	7	0	0

11.	OC 1382	0	0	0	0	0
12.	OC 1388	2	0	2	0	0
13.	OC 1400	0	0	0	0	0
14.	OC 1436	2	1	0	0	0
15.	OC 1458	16	8	9	0	8
16.	OC 1364	0	0	0	0	0
17.	HC_1	0	0	0	0	0
18.	HC_2	2	0	0	0	0
19.	HC_3	0	0	0	0	0
20.	HC_4	0	0	0	0	0
21.	HC_5	0	0	0	0	0
	**TOTAL**	**33**	**13**	**20**	**0**	**12**

REFERENCES

1. Adams, D.L.; Adams, D.K.; He, J.; Kalhor, N.; Zhang, M.; Xu, T.; Komaki, R. Sequential tracking of PD-L1 expression and RAD50 induction in circulating tumor and stromal cells of lung cancer patients undergoing radiotherapy. *Clinical cancer research*, **2017**, *23*, 5948–5958.
2. Adams, D.L.; Alpaugh, R.K.; Tsai, S.; Tang, C.-M.; Stefansson, S. Multi-Phenotypic subtyping of circulating tumor cells using sequential fluorescent quenching and restaining. *Scientific reports*, **2016**, *6*, 33488.
3. Cherian, S.; Levin, G.; Lo, W.Y.; Mauck, M.; Kuhn, D.; Lee, C.; Wood, B.L. Evaluation of an 8-color flow cytometric reference method for white blood cell differential enumeration. *Cytometry B Clin Cytom*, **2010**, *78*, 319–328, doi:10.1002/cyto.b.20529.
